# Supplementary material for: Celestial compass sensor mimics the insect eye for navigation under cloudy and occluded skies
Source: Commun Eng. 2023 Nov 15;2:82. doi: 10.1038/s44172-023-00132-w (PMC10955862; doi:10.1038/s44172-023-00132-w)
Supplement: Supplementary file 2 — Supplementary Information [file 44172_2023_132_MOESM2_ESM.pdf]

Supplementary Information

Celestial compass sensor mimics the insect eye for navigation under cloudy and occluded skies

Evripidis Gkanias[1*], Robert Mitchell[1], Jan Stankiewicz[1], Sadeque R. Khan[2], Srinjoy Mitra[3], and Barbara Webb[1]

[1]School of Informatics, University of Edinburgh, Edinburgh, UK
[2]School of Engineering and Physical Sciences, Heriot-Watt University, Edinburgh, UK
[3]School of Engineering, University of Edinburgh, Edinburgh, UK
[*]Corresponding author: ev.gkanias@gmail.com

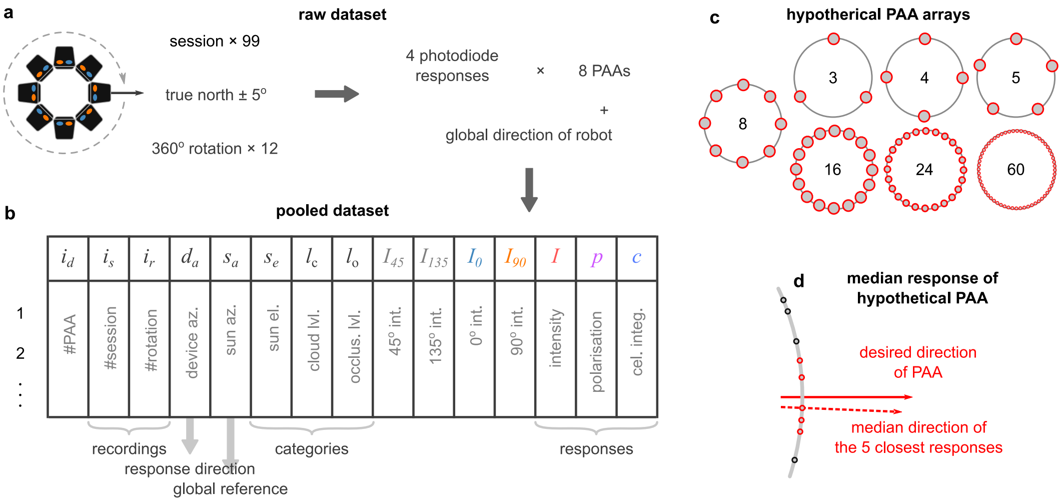

Supplementary Figure 1. The data collection and transformation process. (**a**) Using our designed robot, 99 sessions were recorded in Italy and South Africa under different weather conditions and occlusion with trees and buildings. In each session, the robot was initialised approximately north (±5°), it took a snapshot of the sky for reference (using a camera with a fish-eye lens), and began a complete (360°) rotation on the spot, logging its global direction (relative to the starting point) and the responses of the four photoreceptors from each of the eight polarisation axis analysers (PAAs). (**b**) The collected data were pooled in a dataset (two-dimensional matrix), where we stored the identity of the PAA, session, and rotation, the azimuths of the PAA and sun, the elevation of the sun, the cloud and occlusion category (estimated by eye using the snapshot of the sky at the beginning of each session), the responses of all the photoreceptors and PAAs, and the extracted intensity, polarisation, and their celestial integration. (**c**) Using this technique, we could create sensors with any number of hypothetical PAAs and compare their performance. (**d**) To calculate the response of I, p and c in an arbitrary direction, we used the recorded response of a PAA that faced the median direction of the 5 closest recorded directions to the desired one in a specific rotation.

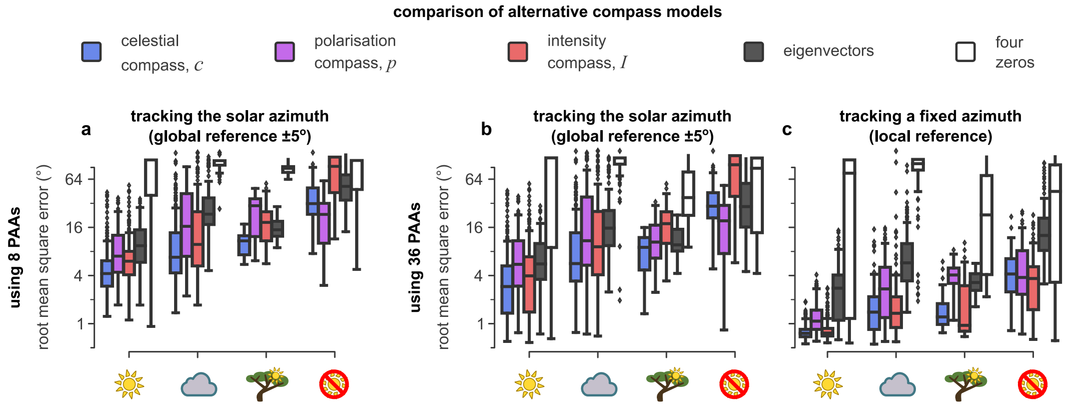

Supplementary Figure 2. Performance of alternative models for different spatial resolutions.
The boxes represent the distribution of root mean square error (RMSE) across the subset of the data where the sky was almost clear (sun), with thick clouds (cloud), with severe occlusions (tree), or where the sun was completely covered by clouds or canopies. Blue represents the 'celestial compass', purple represents the 'polarisation compass', red represents the 'intensity compass', dark grey represents the 'eigenvectors', and white represents the 'four zeros' models. We report the RMSE of predicting (**a**) the solar azimuth when using 8 polarisation axis analysers (PAAs). (**b**) Using 36 PAAs to calculate the respective RMSEs of the solar azimuth, or (**c**) any fixed azimuth. The data shown are for solar elevations of at least 15°. Box-plot: centre line, median; box limits: upper and lower quartiles; whiskers: 1.5× interquartile range; points: outliers. Vertical axes are in the $\log_2$ scale. Ticks without labels denote (from bottom to top) 0°, 2°, 8°, 32°, and 90° RMSE.

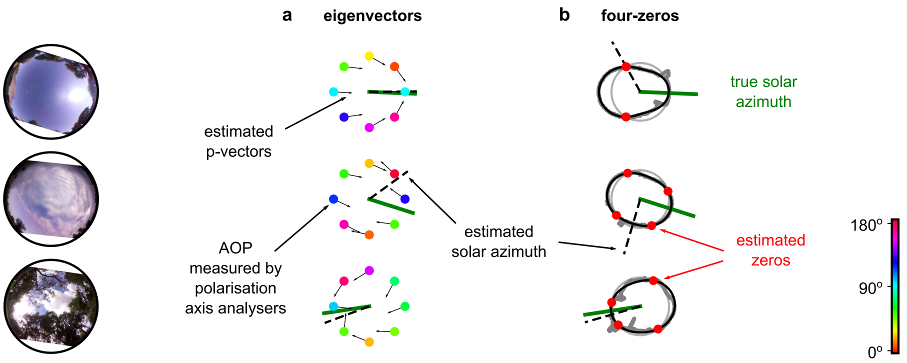

Supplementary Figure 3. Examples from alternative compass models.
(left) Images showing the sky condition during the specific experiments. (**a**) The predictions of the eigenvectors model. The coloured dots show the positions of polarisation axis analysers (PAAs), their colour denotes the angle of polarisation (AoP) estimated using equation (6) (see methods), and the arrows represent the polarisation vectors (**p**) as calculated by equation (9) (see methods). (**b**) The predictions of the four zeros model. In grey, we plot the actual polarisation responses (p) of the PAAs, in black are the fitted lines described by equation (14) (see methods), in red are the estimated four zeros, in dashed black is the predicted solar meridian from the model, and in solid green is the actual solar meridian. The predictions were estimated using only 8 analysers.

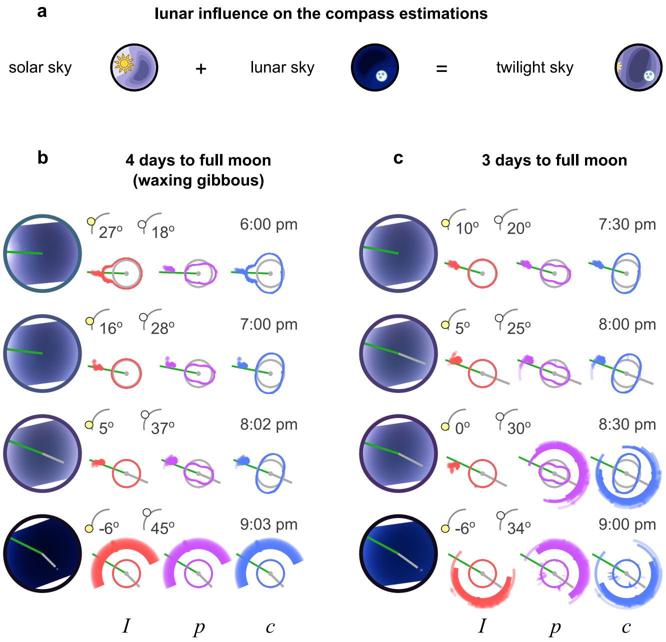

Supplementary Figure 4. Influence of the lunar sky on the celestial compass predictions.
(**a**) Schematic of the twilight sky, which mixes the solar sky and the lunar sky. (**b**) Examples of sky and responses of the different optical processing units (intensity, I, polarisation, p, and celestial integration, c) on May 12 (2022) when it was four days before the full moon (waxing gibbous phase). (**c**) Examples of sky and responses of the different optical processing units on May 13 (2022) when it was three days before the full moon. Data were collected in Sardinia and the full moon was on May 16, 2022. Next to each fish-eye image of the sky condition, we report the solar elevation, time of the day, responses and predictions of the different optical processing units and compasses. Red represents the intensity, purple is the polarisation, and blue is the celestial integration. Each example represents a session where the twelve arrows and circular lines represent the different rotations of the form of the median and quartiles respectively (we used 360 samples per rotation, homogeneously distributed in a circle).

Supplementary Table 1. Performance of different numbers of analysers.

RMSE	number of PAAs					
	**3-5**	**6-7**	**8**	**9-19**	**20-35**	**36-60**
Global	9.49°	5.45°	4.26°	3.37°	2.86°	2.68°
Local	8.10°	3.78°	2.74°	1.67°	0.74°	0.41°

RMSE: the median root mean square error (RMSE) across all the sessions and rotations in a group. Global: the RMSE with respect to the global reference (solar meridian). Local: the RMSE with respect to the local reference (starting point or rotation). PAA: polarisation axis analyser.

Supplementary Table 2. Performance in different solar elevations.

RMSE	solar elevation				
	≤0°	5°	10°	15° - 80°	85°
Global	104.21°	12.83°	8.66°	4.31°	29.30°
Local	102.00°	10.03°	5.68°	2.87°	1.36°

RMSE: the median root mean square error (RMSE) across all the sessions and rotations in a group. Global: the RMSE with respect to the global reference (solar meridian). Local: the RMSE with respect to the local reference (starting point or rotation).

Supplementary Table 3. Overall performance in different locations.

**RMSE**	**Sardinia**	**Vryburg**	**Bela Bela**
Global	4.42°	4.42°	4.78°
Local	2.88°	2.53°	2.42°

RMSE: the median root mean square error (RMSE) across all the sessions and rotations. Global: the RMSE with respect to the global reference (solar meridian). Local: the RMSE with respect to the local reference (starting point or rotation).

Supplementary Table 4. Performance with cloudy skies.

RMSE	type of clouds			
	**clear**	**thin / broken**	**thick solid**	**thick uniform**
Global	4.37°	4.87°	17.73°	28.26°
Local	2.79°	2.57°	5.42°	7.13°

RMSE: the median root mean square error (RMSE) across all the sessions and rotations. Global: the RMSE with respect to the global reference (solar meridian). Local: the RMSE with respect to the local reference (starting point or rotation). Types of clouds: clear = no clouds; thin / broken = thin broken clouds, thick broken clouds, mixed broken clouds, thin solid clouds, and thin uniform clouds.

Supplementary Table 5. Performance with occlusions.

RMSE	no occlusion	location of occlusion			
		far from trees	close to trees	dense trees	dense side
			**8 PAAs**		
Global	4.26°	4.55°	9.92°	11.50°	5.73°
Local	2.80°	2.51°	6.14°	5.42°	2.41°
			**16 PAAs**		
Global	2.95°	3.67°	8.34°	10.28°	5.56°
Local	1.27°	1.66°	2.89°	2.26°	1.48°

RMSE: the median root mean square error (RMSE) across all the sessions and rotations. Global: the RMSE with respect to the solar azimuth. Local: the RMSE with respect to a fixed azimuth. Dense occlusions on one side of the view include both cases with trees and with buildings. PAA: polarimeter axis analyser.

Supplementary Table 6. Performance of alternative models.

RMSE	sky condition	model				
		celestial compass	pol. compass	intensity compass	four zeros	eigen-vectors
				**8 PAAs**		
Global	clear	4.28°	7.01°	6.07°	96.73°	9.43°
	cloudy	6.80°	16.32°	9.77°	98.72°	23.05°
	occluded	10.81°	29.26°	18.25°	84.23°	14.80°
	no-sun	31.07°	22.88°	89.26°	93.74°	50.63°
Local	clear	2.58°	3.76°	3.34°	80.26°	8.03°
	cloudy	3.77°	9.42°	3.95°	95.98°	17.63°
	occluded	6.42°	24.12°	4.68°	77.38°	9.48°
	no-sun	9.87°	8.68°	9.22°	61.68°	39.53°
				**36 PAAs**		
Global	clear	2.83°	5.31°	3.83°	99.12°	5.38°
	cloudy	5.43°	10.37°	8.74°	101.90°	14.84°
	occluded	8.60°	9.99°	16.84°	35.23°	9.23°
	no-sun	27.61°	18.25°	89.27°	80.91°	27.30°
Local	clear	0.59°	1.09°	0.60°	70.38°	2.70°
	cloudy	1.45°	2.65°	1.41°	92.12°	5.54°
	occluded	1.26°	3.93°	0.91°	21.57°	3.17°
	no-sun	4.04°	3.66°	3.55°	41.97°	12.05°

RMSE: the median root mean square error (RMSE) across all the sessions and rotations. Global: the RMSE with respect to the solar azimuth. Local: the RMSE with respect to a fixed azimuth. PAA: polarisation axis analyser.

Supplementary Table 7. Polarisation axis analyser and robot components.

Device	Part	
PAA	linear polarizer	OU2525, KO
	UV photodiode	SG01D-18, SGLUX
	power management	ADP7118-2.5V, ADP7142-5V
	LT	LTC6082
	ADC	ADS112C04, TI
	IO output	6-pin JST-GH
Robot	Kit	TurtleBot3 (Burger), ROBOTIS
	IMU / MPU	MPU-9250 (Embedded in the Turtlebot OpenCR board), TDK
	Camera	B0103 (Raspberry Pi Camera), Arducam
	Lens 1	LN008, Arducam
	Lens 2	LN020, Arducam
	I^2C multiplexer	TCA9548A, Adafruit
	I^2C breakout	Custom, available in Supplementary Data S1
	Software	Ubuntu 20.04 (laptop)<br>Raspbian 9 (Stretch) (robot)<br>ROS1 Noetic (laptop)<br>ROS1 Kinetic (robot)<br>OpenCV (Version 2.4.9)

PAA: polarisation axis analyser, KO: Knight Optical, UV: ultraviolet, LT: linear technology, ADC: analogue-to-digital converter, TI: Texas Instruments, IO: input/output, JST: Japan solderless terminal, IMU: inertia measurement unit, MPU: motion processing unit, TDK: Tokyo Denki Kagaku Kōgyō K.K., ROS: robot operating system, I^2C: inter-integrated circuit.
