# Supplementary material for: Celestial compass sensor mimics the insect eye for navigation under cloudy and occluded skies
Source: Commun Eng. 2023 Nov 15;2:82. doi: 10.1038/s44172-023-00132-w (PMC10955862; doi:10.1038/s44172-023-00132-w)
Supplement: Supplementary file 3 — Description of Additional Supplementary Files [file 44172_2023_132_MOESM3_ESM.pdf]

Description of Additional Supplementary Files

File name: Supplementary Data 1

Description: Computer-aided design (CAD) files of the sensor. The standard tessellation language (STL) files that were required to 3D print the housing of the designed sensor. All measurements are in millimetres. The ‘I2C Breakout’ directory contains the Autodesk EAGLE project files for the custom inter-integrated circuit I2C breakout board used for interfacing with the Adafruit TCA9548A I2C multiplexer. The ‘TCA_Grove_Interface_Board’ directory contains the files required to reproduce the board on a printed circuit board (PCB) mill.
